# Genome analyses of *bla*_NDM-4_ carrying ST 315 *Escherichia coli* isolate from sewage water of one of the Indian hospitals

**DOI:** 10.1186/s13099-018-0247-8

**Published:** 2018-05-24

**Authors:** Ayesha Z. Beg, Asad U. Khan

**Affiliations:** Medical Microbiology and Molecular Biology, Laboratory Interdisciplinary, Biotechnology Unit, Aligarh Muslim University, Aligarh, 202002 India

**Keywords:** NDM-4, ST 315, Pathogenic, ExPECs, Hospital setting

## Abstract

**Background:**

Emergence of carbapenem resistant *Escherichia coli* pathovars and their environmental dissemination are alarming problems. *E. coli* isolated from sewage water of hospital setting conferred a high resistance towards β-lactams, particularly towards carbapenem. This prompted us to perform whole genome sequence analysis to investigate the antimicrobial determinants, pathogenicity status and mobile genetic elements associated with resistance genes.

**Results:**

To the best of our knowledge this is the first report of ST 315 carrying NDM-4 from India. The genome analysis has revealed the unknown characteristics associated with this sequence type (ST 315) like resistance and virulence factors. Based on virulence markers, its pathotype was identified as ExPEC. Furthermore, a mobile plasmid with multiple β-lactamases genes and clinically relevant resistance markers was detected. Phylogenetic analysis of Inc F plasmids sequences carrying ESBLs and NDM variants, revealed un-relatedness in these plasmids due to their varying size and backbone sequences.

**Conclusions:**

Presence of carbapenem resistant *E. coli* ST 315 with high level antibiotic resistance, near hospital environment is an alarming situation in context to its spread. WGS based analyses have provided details on virulence and resistance status which could overcome the lack of information available on ST 315, globally. This could further help in its quick detection and control in clinical settings.

**Electronic supplementary material:**

The online version of this article (10.1186/s13099-018-0247-8) contains supplementary material, which is available to authorized users.

## Background

*Escherichia coli* share a commensal relationship with humans and animals. The extensive acquisition of virulence genes has potentiated *E. coli* to become pathogenic [1]. The *E. coli* pathotypes are identified on the basis of virulence determinants present in the genome. A pathotype, ExPEC (extra intestinal pathogenic *E. coli*) has been reported in extra intestinal, neonatal meningitis and septicaemia infections. Over the years ExPECs are being increasingly recognised for plasmid-mediated carriage of extended spectrum β-lactamases and carbapenemases (metallo β-lactamases, MBLs) [2]. The emergence of a carbapenemase, New Delhi-Metallo β-lactamase (NDM-1) has conferred resistance to last resort β-lactams which has further made the management of ExPECs difficult [3]. A single amino acid variation (Met154Leu) in NDM-1 has resulted in the emergence of a novel NDM-4 which has extended and increased hydrolytic activity towards β-lactams, especially towards carbapenem [4].

The bacterial isolate AK-1 found in hospital sewage water was subjected to antibiotic susceptibility testing which revealed an exorbitantly elevated MIC values against β-lactams [5]. This unusual resistance in AK-1 strain intrigued us to further explore other genetically predisposed features through whole genome sequencing.

## Materials and methods

### *E. coli* isolation and characterisation

The isolate for whole genome sequence analysis was collected and identified as reported in our previous published study [5]. It was identified and characterised as NDM-4 producing *E. coli* strain.

### Whole genome sequencing, annotation and analysis

The bacterial DNA was isolated from the AK-1 strain by Qiagen’s QIAamp DNA mini kit and GE SimpliNano UV–Vis Spectrophotometer was used to measure the concentration and purity of the DNA. The genomic DNA was subjected to whole genome sequencing on Illumina NextSeq 500 platform using the paired-end 2 × 150 nt sequencing protocol. The raw sequence data was further analysed by FastQC tool for quality control purposes [6]. SPAdes version 3.10.1 was used to create denovo assembly with genome coverage of 266.181× [7]. Genome annotation was performed by NCBI Prokaryotic Genome Annotation Pipeline using Best-placed reference protein set and GeneMarkS+ methodology.

StringMLST tool [8] was used to determine sequence type and multi-locus sequence typing database of *E. coli* at PubMLST (http://ukmirror1.pubmlst.org/databases.shtml) [9] was used as reference. The serotype of the strain (fliC, wxy and wzx genes) was determined by SerotypeFinder [10]. Resistance genes were identified using CARD and ARDB databases [11, 12]. Virulence factors were determined using the combination of UniProt and Virulence finder database (VFDB) [13]. Further genome analyses was done using ISfinder [14] and RAC [15]. Phylogenetic analysis was done on MEGA (version 7) using plasmid sequences retrieved from GenBank.

### Quality assurance

*Escherichia coli* ST 315 genomic DNA was extracted from a single colony and this strain was maintained. The 16s rRNA gene from the draft genome was confirmed to check for contamination. High quality of reads were confirmed by CLC bio Genomic Workbench version 9 (CGWB) and selected for assembly.

## Results and discussion

### General features

The genome NSBV00000000 *E. coli* consists of 129 contigs, which equals to 5,076,053 bp in length. The mean G+C content of the genome is 50.74%. Other features are enlisted in Table 1. The serotype analysis of the AK-1 with the aid of *fliC*, *wzy* and *wzx* genes profile was found to be O7: H15. MLST analysis (*adk*:*fumC*:*gyrB*:*icd*:*mdh*:*purA*:*recA*;4:26:2:25:5:8:19) identified AK-1 as ST 315. AK-1, *E. coli* ST 315 is included in clonal complex ST38 which is associated with phylogenetic group D and this clonal complex has preference to harbour ESBLs, particularly CTX-M-14 and CTX-M-15 [16]. Ewers et al. described numbers of *E. coli* ST 315 isolates carrying *bla*_NDM-1_ and globally associated with human host [17].

**Table 1:** General features of AK-1 genome

Feature	Number
Genes (total)	5271
CDS (total)	5176
Genes (coding)	4948
CDS (coding)	4948
Genes (RNA)	95
rRNAs	6, 2, 5 (5S, 16S, 23S)
Complete rRNAs	6, 1 (5S, 16S)
Partial rRNAs	1, 5 (16S, 23S)
tRNAs	71
ncRNAs	11
Pseudo genes (total)	228
CRISPR arrays	2

AK-1 was found to carry a plasmid of 155,678 bp (Additional file 1: Figure S1). pMLST identified plasmid belonging to IncF group having alleles, repFIB and repFII.

## Resistance genes

**Fig. 1:**
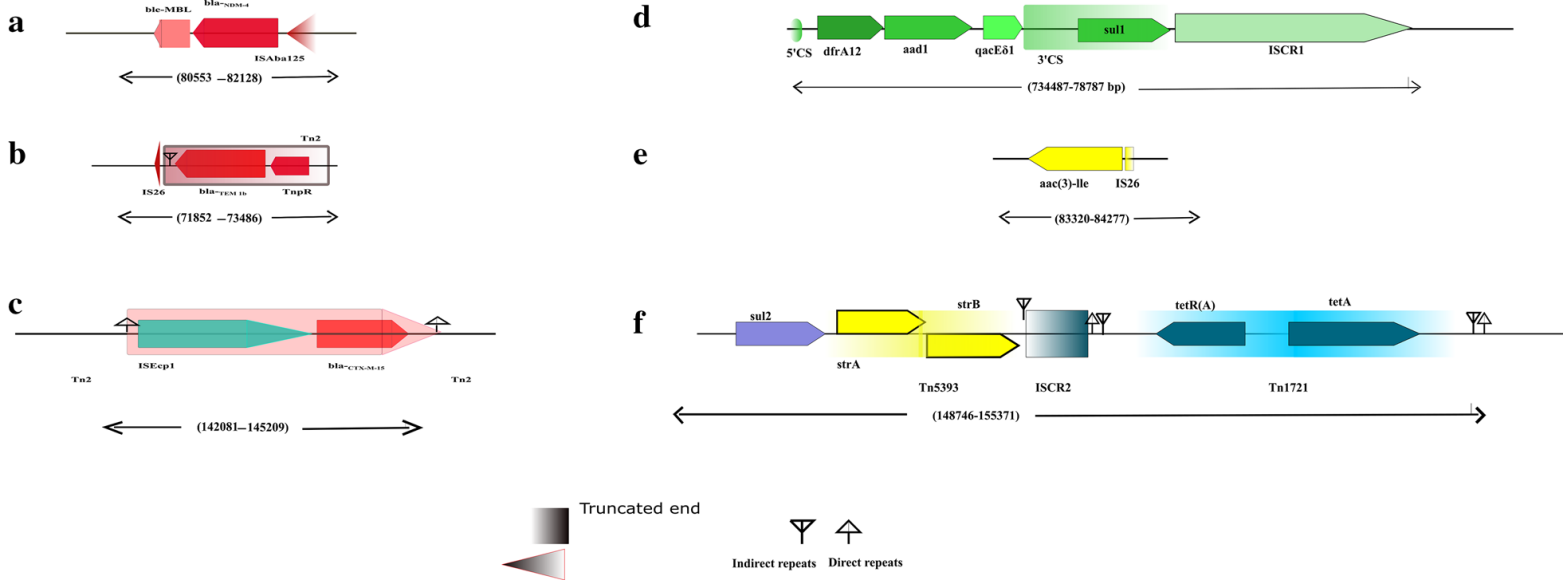
Genetic context of resistance genes present in pAK-1. **a** *bla*_NDM-4_, **b** *bla*_CTX-M-15_, **c** *bla*_TEM1b_, **d** Class I integron carrying: *dfrA12, aad, qacEδ1*and *sul1*, **e** aac(3)-Ile, **f** *sul2*, *strA*, *strB*, *tetR*(A) and *tetA*

One of the highlight of this study was an NDM-4 carrying multidrug plasmid. It harboured 15 resistance markers (Additional file 2: Table S1**)** and some of them were in association with mobile genetic elements (MGEs). Genetic context of the resistance markers on the plasmid was determined to assess their association with mobile genetic elements and their potential for horizontal gene transfer. Genetic environment of *bla*_NDM-1_ have revealed the downstream presence of *ble*_MBL_ and upstream presence of remnants of, or entire ISAba125 [18]. The plasmid pAK1 shared similar arrangement for *bla*_NDM-4_ with partial IS*Aba*125 (Fig. 1a).

The genetic environment of *bla*_TEM1b_ was identified to be flanked by one truncated copy of IS26. The *bla*_TEM1b_ gene was associated with partial Tn2, it was present distally to tnpR-encoding gene and proximally to an IR (Fig. 1b) [19].

A truncated Tn2 transposon unit was found to be associated with *bla*_CTX-M-15_. It was revealed that both DRL and DRR were bracketing this unit where ISEcp1 was distally located to *bla*_CTX-M-15_ gene (Fig. 1c). This arrangement is very common and ISEcp1 is reported to mediate the mobilization of *bla*_CTX-M-15_ [20, 21].

Other previously reported resistance genes and associated mobile genetic elements were found on AK-1 plasmid. (i) Class I integron carrying *qacE∆ 1*, *sul 2*, *dfrA12* and *aadA2* (Fig. 1d) [22], (ii) *rmt B* was found to be associated with partial sequence of IS 26 (Fig. 1e) and upstream to *bla*_TEM1b_ (not shown) [23], (iii) *sul1* gene upstream to *strA* and *strB* genes which are bracketed by Tn 5903 [24], (iv) *tet (A)* efflux protein and its regulator *tet R (A)* associated with Tn1721 mobile element [25].

AK-1 chromosome carries wide range of resistance markers towards major classes of antibiotics like flouroquinoles, macrolides, aminoglycosides, tetracycline, trimethoprim isoniazid, triclosan, elfamycin and β-lactams (Additional file 3: Table S2). Extensive numbers of genes conferring resistance towards β-lactams were found; four types of penicillin binding proteins and class C β-lactamases, *bla*_CFE 1_ and *bla*_PEDO 2_. Accumulative effect of these genes explains the high level of phenotypic resistance towards β-lactams in AK-1 [5].

## Virulence factors of ST 315 *E. coli*

ST 315 *E. coli* has been reported earlier in urosepsis, intra-abdominal infections and primary sepsis in medical cases [16]. Therefore, comparative analysis [with NC_017646 (NMEC), NC_008253 (UPEC), NC_017631 (UPEC), NC_007946 (UPEC)] and exploration of virulence genes in AK-1 was performed which resulted in identification of assorted virulence factors. These are commonly associated with ExPEC isolates [26] as shown in Table 2. *E. coli* type III secretion system 2 (ETT2) identified in AK-1 has been previously reported in *E. coli* strains in partial or complete form [27]. ETT2 is associated with virulence regulation in some ExPEC strains and pathogenicity in septicemic *E. coli* [28]. Pathogenicity island (PAI), type 6 secretion system was identified in AK-1 [29]. Multiple PAI, invested in fimbriae and adhesions expression, were observed in AK-1 strain which are described as (i) Type 1 fimbriae is common in UPEC, causes infection in mucous surfaces by inducing adhesion and virulence [30], (ii) Chaperone usher (CU) fimbriae clusters *yad* and *sfm* provide additional adhesion to the host [31], (iii) Mat (meningitis associated and temperature regulated) fimbria or *E. coli* common pilus (ECP) responsible for colonisation and adherence in host [32], (iv) Curli fibres binds to hosts matrix and plasma protein, and is reported to cause haemagglutination, fibronectin binding and formation of proteolytically active plasmin which aids in bacterial diffusion through tissue disintegration [30], (v) ExPEC specific FdeC (factor adherence *E*. *coli*) responsible for bacterial fitness and colonization in UTI [33].

**Table 2:** Virulence genes

Virulence factors				
Adhesins/fimbriae	Iron uptake	Toxins	Secretion systems	Protectins/invasins
Type 1 fimbriae NSBV01000003 (15138–21501) sfm fimbriae NSBV01000011 (7740–14015) yad fimbriae NSBV01000005 (66168–72121) MAT(ECP) fimbriae NSBV01000007 (119869–126709) FdeC NSBV01000007 (106372–110622) Curli NSBV01000004 (93069–97942)	ChuA NSBV01000028 (31777–40728) Enterobactin NSBV01000021 (27676–46582) Aerobactin NSBV01000005 (59212–52929)	HlyA NSBV01000011 (54096–55271) HlyE NSBV01000026 (46866–47777)	TypeIII secretion system (ETT2) NSBV01000002 (15607–31153) TypeVI secretion system NSBV01000032 (5165–28659)	ColV NSBV01000002 (84476–84964) ibeB NSBV01000021 (57575–58957) K1capsule NSBV01000002 (150300–158286) ompA NSBV01000004 (166414–167466)

Haemolytic toxins were also identified in AK-1 (i) Hemolysin α is associated with ExPEC virulence and attacks immune cell [34], (ii) membrane pore-forming toxin HlyE lyses mammalian cells and erythrocytes [35].

ExPECs cope up with low iron availability by secreting siderophores which retrieves sequestered iron from host proteins [36]. AK-1 was identified to harbour aerobactin, enterobactin and chuA siderophores. Proectins/invasins like ibeB, ompA and K1 capsule have been reported in invasion of brain microvasular endothelial cells [37].

## Phylogenetic analysis of plasmid

**Fig. 2:**
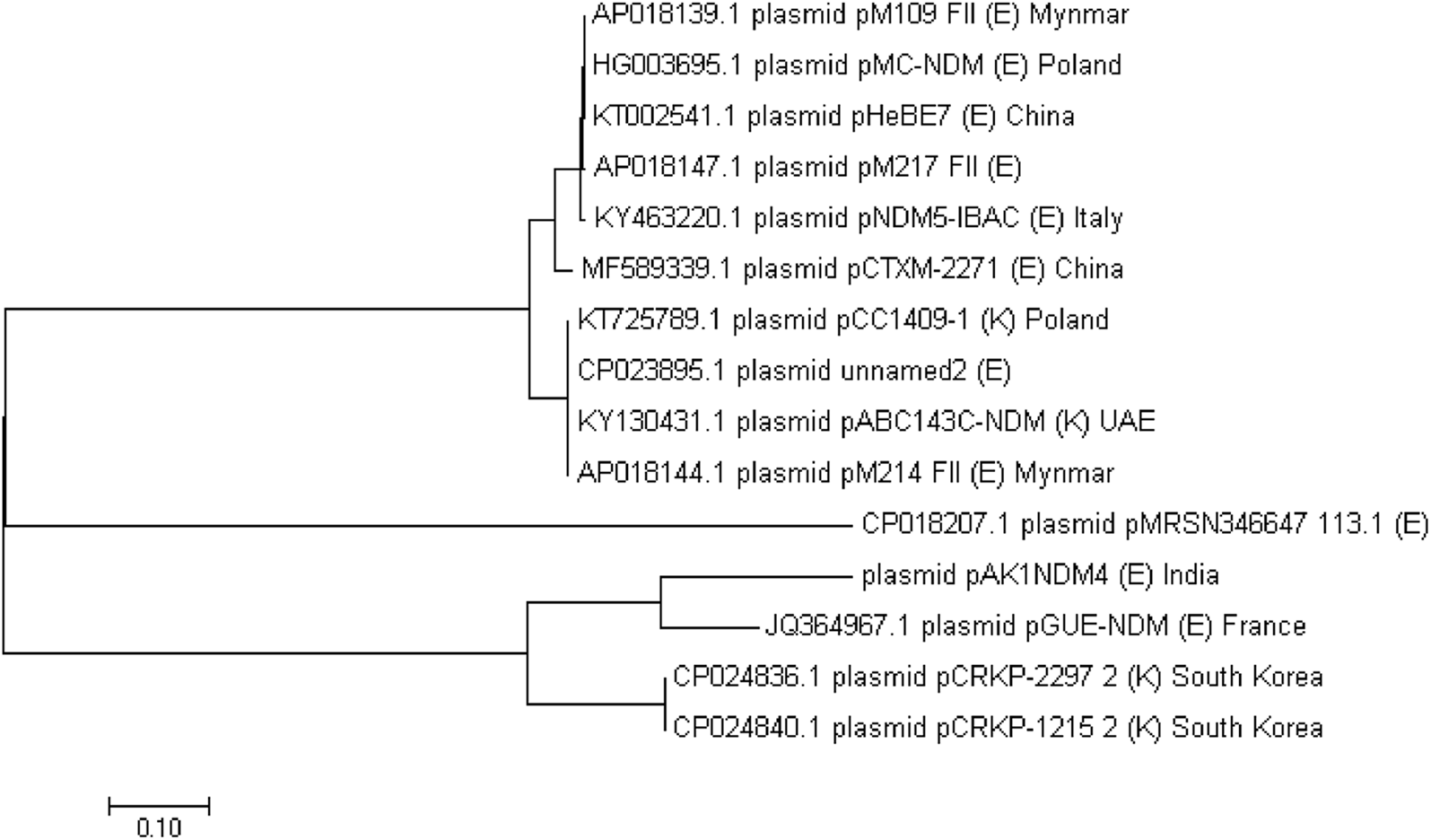
Phylogenetic tree of Inc F plasmids. Based on Inc F plasmids (having repFIB and/or repFII alleles) sequences, tree was generated using MEGA 7. GenBank accession ids of the plasmids and location of their identification are shown here. E and K are abbreviation for hosts, *Escherichia coli* and *Klebsilla pneumonia*

Presence of emerging NDM variant along with multidrug resistances on a mobile Inc F plasmid prompted us to compare the pattern of their dissemination and relatedness. Phylogenetic analysis was performed using plasmid sequences which produced significant alignment with AK-1 plasmid, and the query coverage was found between 44 and 53% of all the plasmid sequences with ~ 99% identity. Furthermore, plasmid sequences having alleles, repFIB and/or repFII and acquired genes, *bla*_TEM1_ and/or *bla*_CTX-M-15_, and/or *bla*_NDM_ variants (NDM-4/NDM-1/NDM-5), were specifically selected for comparisons with AK-1 plasmid (Fig. 2). The result showed overall dissimilarity in the backbone sequences except for plasmid pGUE NDM (France) which differs by nine single nucleotide variations with AK-1 plasmid. Distribution and backbone sequences of Inc F plasmids harbouring NDM variants and/or ESBLs (CTX-M-15/TEM1b) are inconsistent. Active mobilome could account for such high variation in plasmid sequences. This implies ExPECs carrying such plasmids could have fluctuating resistance profile leading to a concern for clinicians.

The genetic context of NDM-4 in AK-1 plasmid was similar to plasmids (pM109 FII, pMC-NDM, pGUE NDM, pCRKP-2297, pCRKP-1215, pM214 FII, and pNDM5-IBAC) carrying NDM variants. The genetic environment for *bla*_NDM_ remained conserved in these plasmids. This suggests that alteration in *bla* gene originated new NDM variants. Furthermore, genetic context of *bla*_TEM1b_ and *bla*_CTX-M-15_ were almost similar in these plasmids.

## Conclusions

Presence of plasmid harbouring *bla*_NDM-4_ and other β-lactamase genes near the hospital setting environment is a serious concern in context to its circulation and spread in hospital settings. It is the first time NDM-4 producing ST 315 *E. coli* was detected in India. Moreover, no genome based information on ST 315 strain is yet available. Lack of information on mechanism of virulence, transmission sources and other genetic characteristics have become an impasse for alleviation of ExPECs infections. AK-1 genome based virulence profile provides cause of serious infections by ST 315 ExPEC, a common microflora of healthy individuals. Genome informed virulence and resistance mechanisms will definitely help in identification of this ExPECs in healthcare settings and controlling the clonal spread of carbapenamase carrying *E. coli* pathovars.

## Additional files

**Additional file 1: Figure S1.** Linear map of plasmid AK-1.

**Additional file 2: Table S1.** Resistance genes associated with plasmid.

**Additional file 3: Table S2.** Chromosomal encoded resistance genes.
