# Supplementary figures and images for: Genome analyses of blaNDM-4 carrying ST 315 Escherichia coli isolate from sewage water of one of the Indian hospitals
Source: Gut Pathog. 2018 May 24;10:17. doi: 10.1186/s13099-018-0247-8 (PMC5968484; doi:10.1186/s13099-018-0247-8)

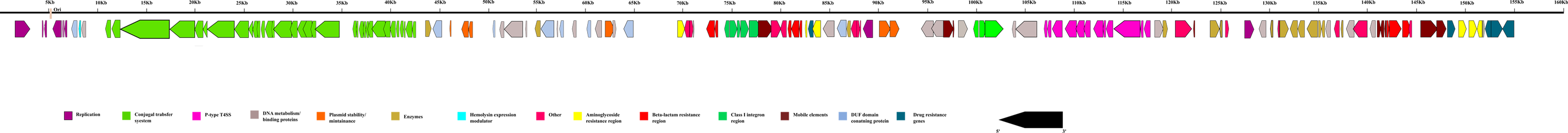

Supplement: Supplementary file 1 — Additional file 1: Figure S1. Linear map of plasmid AK-1. [file 13099_2018_247_MOESM1_ESM.tif]
