# Supplementary material for: Genome analyses of blaNDM-4 carrying ST 315 Escherichia coli isolate from sewage water of one of the Indian hospitals
Source: Gut Pathog. 2018 May 24;10:17. doi: 10.1186/s13099-018-0247-8 (PMC5968484; doi:10.1186/s13099-018-0247-8)
Supplement: Supplementary file 2 — Additional file 2: Table S1. Resistance genes associated with plasmid. [file 13099_2018_247_MOESM2_ESM.docx]

**Table S1**. Resistance genes associated with plasmid

| **RESISTANCE GENES IN PLASMID** | | |
| --- | --- | --- |
| **Resistance genes** |  | **Reference sequences** |
| *bla* _NDM-4_  *bla*_CTX-M-15_  *bla*_TEM-b1_  strB  strA  aacC2  aadA2  aac(2')-Ia  tet R (A)  tet (A)  sul2  sul1  ble_MBL_  qacE delta 1  dfrA12 | Subclass B-1 β-lactamase  Class A β-lactamase  Class A β-lactamase  aph6id (Aminoglycoside O-phosphotransferase)  aph33ib (Aminoglycoside O-phosphotransferase)  Aminoglycoside acetyltransferase  ANT(3'')-Ia family aminoglycoside nucleotidyltransferase  Aminoglycoside  N-acetyltransferase  TetR family transcriptional regulator  Tetracycline efflux MFS transporter  Sulphonamide resistant dihydropteroate synthase  Sulfonamide-resistant dihydropteroate synthase  Bleomycin resistance genes.  Quaternary ammonium compound efflux SMR transporter  Dihydrofolate reductase | NSBV01000047  (7436-8248)  NSBV01000054  (2348-3223)  NSBV01000068  (148-1008)  NSBV01000045  (5293-6129)  NSBV01000045  (4490-5293)  NSBV01000063  (914-1774)  NSBV01000047  (988-1779)  NSBV01000001  (129608-130069)  NSBV01000045  (7363-8040)  NSBV01000045  (8119-9318)  NSBV01000045  (3614-4429)  NSBV01000045  (2284-3123)  NSBV01000045  (7067-7432)  NSBV01000045  (1943-2290)  NSBV01000045  (83-580) |
