# Supplementary material for: Genome analyses of blaNDM-4 carrying ST 315 Escherichia coli isolate from sewage water of one of the Indian hospitals
Source: Gut Pathog. 2018 May 24;10:17. doi: 10.1186/s13099-018-0247-8 (PMC5968484; doi:10.1186/s13099-018-0247-8)
Supplement: Supplementary file 3 — Additional file 3: Table S2. Chromosomal encoded resistance genes. [file 13099_2018_247_MOESM3_ESM.docx]

Table S2. **Chromosomal encoded resistance genes.**

| **DRUG CLASSES** | **RESISTANCE GENES/REFERENCE SEQUENCE** |
| --- | --- |
| **β-lactams** | bla__PEDO-2_  NSBV01000004  (201996-202643  omp36  NSBV01000039  (13942-15135)  pbp1c  NSBV01000006  (78105-80417)  pbp2a  NSBV01000022  (8851-10752)  pbp1a  NSBV01000010  (101820-104372)  pbp1B  NSBV01000005  (55338-57872)  pbp4b  NSBV01000006  (169347-170648)  *bla_cfe1*  NSBV01000008  (160246-161379) |
| **Aminoglycosides** | ksgA  NSBV01000005  (169452-170273)  rpsL  NSBV01000010  (147357-147731)  rmtB  NSBV01000061  (100-855) |
| **Tetracyclines** | tet34  NSBV01000005  (80203-80739) |
| **Macrolide** | oleC  NSBV01000005  (78465-79391) |
| **Flouroquinole** | gyrA  (174894-177521)  qnrS1  NSBV01000048  (5883-6539)  mfd  NSBV01000004  (24100-27546) |
| **Trimethoprim** | dfrA14  NSBV01000090  (261-743) |
| **Peptide Antibiotics** | arnA  NSBV01000001  (141130-143118)  bacA  NSBV01000002  (276276-277097)  rpoC  NSBV01000036  (18508-22731) |
| **Isoniazid and Triclosan** | fabI  NSBV01000014  (98672-99460) |
| **Fluoroquinole and Aminocoumarin** | parC  NSBV01000002  (233315-235573) |
| **Elfamycin** | EFTu mutant  NSBV01000010  (150580-150866)  EFTu mutant  NSBV01000083  (1-956) |
